# Discriminant Analysis of Brazilian Stingless Bee Honey Reveals an Iron-Based Biogeographical Origin

**DOI:** 10.3390/foods12010180

**Published:** 2023-01-01

**Authors:** Flavia C. Lavinas, Brendo A. Gomes, Marcos V. T. Silva, Renata M. Nunes, Suzana G. Leitão, Mirian R. L. Moura, Rosineide C. Simas, Carla S. Carneiro, Igor A. Rodrigues

**Affiliations:** 1 Programa de Pós-Graduação em Ciências Farmacêuticas, Faculdade de Farmácia, Universidade Federal do Rio de Janeiro, Rio de Janeiro 21941-902, Brazil; 2 Programa de Pós-Graduação em Biotecnologia Vegetal, Universidade Federal do Rio de Janeiro, Rio de Janeiro 21941-902, Brazil; 3 Departamento de Produtos Naturais e Alimentos, Faculdade de Farmácia, Universidade Federal do Rio de Janeiro, Rio de Janeiro 21941-902, Brazil; 4 Laboratorio de Cromatografia e Espectrometria de Massas (LaCEM), Universidade Federal de Goiás, Goiania 74690-900, Brazil; 5 Escola de Engenharia, Universidade Presbiteriana Mackenzie, São Paulo 01302-907, Brazil

**Keywords:** meliponine honey, physicochemical properties, biomes, antioxidant potential, mineral profile, mass spectrometry analysis, chemometrics

## Abstract

Stingless bee honey (SBH) is gaining attention due to its nutritional, sensorial, and medicinal characteristics. This study focuses on the combination of physicochemical properties, antioxidant capacity, mineral profile, and mass spectrometry-based fingerprints, using a chemometric approach to differentiate SBH (*n* = 18) from three different Brazilian biogeographical zones (Caatinga, Cerrado, and Atlantic Forest). The physicochemical properties of SBH varied, resulting in a wide range of water activity, moisture, total soluble solids, pH, and total and free acidity. The Caatinga honey showed the highest and the lowest contents of phenolics and flavonoids, respectively. The antioxidant free-radical scavenging assays demonstrated that the Brazilian SBH has a high antioxidant potential. The mineral profile of honey samples from the Atlantic Forest revealed higher contents of Ca and Fe while the Cerrado and Caatinga honey showed the highest P contents. Partial Least-Squares Discriminant Analysis (PLS-DA) analysis separated the samples into three groups based on the biogeographical zones of harvest. The main separation factors between groups were the *m*/*z* 326 ion and the Fe content. Univariate analysis confirmed that Fe content is important for SBH discrimination. The present results indicate that the origin of SBH can be determined on the basis of mineral profile, especially Fe content.

## 1. Introduction

Brazil is a country of continental dimensions (about 8.5 million km^2^), with marked climatic variations over its territory. Climate zones such as tropical, semi-arid, and temperate areas occur from the north to the south of the country. These climatic differences, allied to the various soil types, lead to major ecological variations with the consequent formation of distinct biomes or biogeographical zones. Brazilian biogeographical zones can be mainly divided into six distinct areas: the Amazon Rainforest; Pantanal (wetlands), Cerrado (Brazilian savannah), Caatinga (semi-arid scrub forest), Pampas (grasslands), and the Atlantic Forest [1]. Unsurprisingly, 70% of the world’s cataloged animal and plant species can be found in this country [2].

Brazilian native meliponines (Apidae, subfamily Meliponinae), or simply stingless bees, are insects of great economic and socioenvironmental importance. These bees play an important role as pollinators of wild botanical individuals, including endemic species, as well as several crops such as passion fruit, Brazilian nut, watermelon, guava, tomato, and açaí [3]. In addition, stingless bees (SB) can produce a distinguished honey with unique compositional and sensory characteristics. Stingless bee honey (SBH) is a high sugar-containing solution often showing higher moisture and free acidity compared to *Apis mellifera* honey [4]. Notably, the physicochemical characteristic of honey is influenced by its complex chemical composition, which may vary according to environmental factors, bee species, botanical resources, storage conditions, and processing methods [5].

Substances bearing phenolic groups are identified as the main factors responsible for the antioxidant capacity of honey. Phenolic substances commonly reported in SBH include phenolic acids (gallic acid, caffeic acid, coumaric acid, ellagic acid, hydroxycinnamic acid) and flavonoids (taxifolin, naringenin, luteolin, quercetin, catechin, apigenin) [6,7]. Other components that may contribute to the antioxidant property of honey are amino acids, proteins, enzymes, carotenoids, and organic acids [8]. Here, we combined physicochemical properties, antioxidant capacity, mineral profile, and mass spectrometry-based fingerprints using a chemometric approach to provide new insights into the influence of biogeographical zones on the compositional singularities of SBH. In addition, the identification of the main factors responsible for honey discrimination would permit the use of these analytes for the investigation of honey origin.

## 2. Materials and Methods

### 2.1. Chemicals

High-Performance Liquid Chromatography (HPLC) grade chloroform, methanol and formic acid were obtained from Tedia Brazil^®^ (RJ, Brazil). Folin-Ciocalteu, ABTS (2,2′-azinobis-(3-ethylbenzothiazoline-6-sulfonic acid), TPTZ (2,4,6-tripyridyl-s-Triazine) and nitric acid were obtained from Sigma-Aldrich (St. Louis, MO, USA). Acetic acid, chloride acid, ferric chloride heptahydrate, iron (II) sulfate heptahydrate, and aluminum chloride hexahydrate were obtained from Dinâmica^®^ (Sao Paulo, Brazil).

### 2.2. Honey Samples

SBH samples (*n* = 18) were provided by the Rio de Janeiro Stingless Beekeepers Association (AME-Rio, Rio de Janeiro, Brazil). The samples were originally collected in different Brazilian biogeographical zones between 2017 and 2018 and stored at low temperature (4 °C) in the dark until the time for the analytical procedures. Table 1 summarizes the SB species and honey origin.

### 2.3. Physicochemical Analysis

The physicochemical properties of SBH, including water activity (Aw), moisture, total soluble solids (TSS), pH, total and free acidity, and hydroxymethylfurfural (HMF), were determined as described by the Association of Official Analytical Chemists [9]. The color features of SBH were determined spectrophotometrically. Initially, the absorbance (Abs) of honey sample solutions (0.5 g honey in 1 mL distilled water) was measured at 635 nm [10]. The results were converted to the Pfund scale (mm) using the equation below. Brown pigment formation was determined by measuring the absorbance (420 nm) of honey samples previously diluted to 4° Brix with distilled water [11].

Pfund = −38.70 + 371.39 × Abs(1)

### 2.4. Total Phenolic (TPC) and Total Flavonoid (TFC) Contents

TPC was determined spectrophotometrically by the Folin-Ciocalteu method [12]. First, 1 g of honey was diluted to 10 mL with distilled water and then filtered through a Whatman^®^ (Maidstone, UK) Grade 1 qualitative filter paper (11 µm). Next, 0.1 mL of this solution was added to 0.5 mL of Folin-Ciocalteau reagent (10% *w*/*v*). After 5 min in the dark, 0.4 mL of a 7.5% sodium bicarbonate solution was added and the mixture was kept in the dark at room temperature for 2 h. The absorbance of the reaction mixture was measured at 760 nm (SpectraMax M5, Molecular Devices, Sunnyvale, CA, USA). Gallic acid was used to produce a calibration curve (7–200 µg/mL), and TPC was expressed as mg gallic acid equivalent per 100 g fresh honey weight (mg GAE/100 g FW). 

TFC was determined by a spectrophotometric assay based on flavonoid-aluminum complex formation [10]. Initially, a honey solution was prepared by mixing 0.25 mL of the sample with 1.25 mL of distilled water and 75 µL of 5% NaNO_2_. Then, 0.15 mL of a 10% AlCl_3_ solution was added and the mixture was allowed to stand for 5 min. After adding 0.5 mL of a 1 M NaOH solution, the final volume was adjusted to 2.5 mL with distilled water. The absorbance of the sample was measured at 510 nm (SpectraMax M5, Molecular Devices, Sunnyvale, CA, USA). Quercetin calibration curves were prepared (50–550 µg/mL) and TFC was expressed as mg quercetin equivalent per 100 g fresh honey weight (mg QE/100 g FW).

### 2.5. Antioxidant Capacity Assays

The radical scavenging capacity of SBH samples was determined by the ABTS method [5]. After the ABTS^•+^ generation step, 2.7 mL aliquots of the radical solution were transferred to test tubes containing samples previously diluted in ethanol (100–500 µg/mL, final concentrations). After 10 min, sample absorbances were measured at 734 nm (SpectraMax M5, Molecular Devices, Sunnyvale, CA, USA). The ABTS^•+^ scavenging capacity percentage was determined as described below.

ABTS^•+^ scavenging capacity (%) = (Abs_cnt_ − A_s_) ×100/A_cnt_ (2)

where Abs_cnt_ = absorbance obtained from the ABTS^•+^ solution; A_s_ = absorbance obtained from samples in the presence of the ABTS^•+^ solution. 

The ferric reducing antioxidant power (FRAP) of honey samples was determined as reported by Benzie & Strain (1996) with some modifications [13]. Initially, the FRAP reagent (2 mL of 10 mM TPTZ solution in 6 N HCl, 2 mL of 20 mM FeCl_3_ solution, and 20 mL of 300 mM acetate buffer, pH 3.6) was warmed to 37 °C prior to analysis. A 20 µL aliquot of honey solution (100 mg.mL^−1^) was added to 180 µL of freshly prepared FRAP reagent, the reaction mixture was incubated at 37 °C for 4 min and absorbance was then measured at 595 nm. A calibration curve was prepared with a ferrous sulfate solution (50–800 µM). FRAP values were expressed as micromoles of ferrous equivalent per 100 g fresh honey weight (μmol Fe^2+^/100 g FW).

### 2.6. Mineral Profile Analysis

The mineral and trace contents of stingless bee honey were determined by Total Reflection X-ray Fluorescence (TXRF). Sample preparation was based on acid digestion (65% HNO_3_) and heat [14]. Blank samples were also prepared to assess possible contamination. Eight replicates were prepared for each sample. Standard solutions (CertiPUR Reference Material, Merck^®^, Rahway, NJ, USA) with different concentrations of well-known elements were prepared and Ga (5.0 µg/g, final concentration) was added as an internal standard. To check the accuracy of the procedure used for quantitative analysis, standard reference material (Bovine liver, NIST^®^, SRM 1577, Gaithersburg, MD, USA) was analyzed, and the calculated data were compared to certified values. An X-ray fluorescence system operating under total reflection conditions was used for sample excitation. For characteristic X-ray detection, a portable automated total reflection X-ray fluorescence system using a low-power X-ray tube and a compact Si-PIN detector was employed [15]. The quantitative analysis of X-ray spectra was performed using the QXAS software 1.2 (International Atomic Energy Agency – IAEA, Seibersdorf, Austria). The mineral content of honey samples was expressed as µg/g fresh weight (FW). 

### 2.7. Mass Spectrometry Analysis

Honey samples were prepared from liquid-liquid partitions using ultra-purified water and chloroform (1:1). The chloroform portion was dried and solubilized in methanol at a concentration of 20 mg/mL. Next, 1 µL of each sample was analyzed with an LCQ Fleet mass spectrometer (MS) with an electrospray source (ESI), operated in positive ionization mode. Samples were analyzed by automated direct injection in 0.1 mL.min−1 flow for 5 min. High purity nitrogen (N2) was used as sheath gas (35 arbitrary units) and auxiliary gas (10 arbitrary units). High purity helium (He) was used as collision gas. The MS parameters were as follows: source voltage 5 kV, source current 100 µA, source temperature 450 °C, capillary voltage 7 V, tube lens voltage 65 V, and capillary temperature 400 °C. MS spectra were acquired with a range of *m/z* 50–1000.

### 2.8. Statistical Analyses

Assays were performed in triplicate, and the results were expressed as mean values with standard deviations (SD). The Shapiro-Wilks test was used to determine the normality of physicochemical, antioxidant, and mineral data (*p* > 0.05). Next, analysis of variance (one-way ANOVA) followed by Tukey’s multiple comparison post hoc test was performed using XLSTAT^®^ software (version 2014, Addinsoft, Paris, France). Mass spectrometry data from each sample were analyzed in XCalibur^®^ 2.2 (ThermoScientific, Waltham, MA, USA), where the one thousand most intense peaks were exported in centroid mode. Variables present in less than 7 samples were discarded to avoid biases. The matrix data consisted of 277 variables that corresponded to mass peak intensities; 13 were physicochemical parameters and 12 were mineral concentrations. Multivariate (PLS-DA) and univariate (One-Way ANOVA and Tukey HSD post hoc test) analyses were carried out using the MetaboAnalyst 5.0 webserver. The Shapiro-Wilks test was used to determine the normality of data. Finally, correlations were established using Pearson’s correlation coefficient (r). The correlations were determined using XLSTAT^®^. A *p*-value < 0.05 was considered significant.

## 3. Results and Discussion

### 3.1. Physicochemical Characterization of SBH

The physicochemical properties of SBH from different biogeographical zones of Brazil are summarized in Table 2. We observed that most samples differed significantly (*p* < 0.05) from one another, even those belonging to the same species and collected in the same region. The variation in the physicochemical properties of SBH has been frequently reported [16], demonstrating that quality standards may be extremely hard to establish.

Water content is considered to be one of the most important features of honey since it affects several properties such as viscosity, specific weight, maturity, flavor, crystallization [17], and microbial growth [18]. Here, SBH displayed Aw values ranging from 0.65 to 0.75. There was a significant difference (*p* < 0.05) between honey samples from the Atlantic Forest and Cerrado zones and between those from the Caatinga and Cerrado zones. Ávila et al., (2019), reported Aw values ranging from 0.77 to 0.91 in thirty-two honey samples of two south Brazilian SB species (*Scaptotrigona* spp. and *Melipona* spp.) [19]. In addition, the Aw values of SBH collected in several cities in the state of Santa Catarina (SC, south Brazil) ranged from 0.67 to 0.78 [20]. The *Tetragonisca angustula* honey collected in the municipality of Piracicaba (SP, Southeast Brazil) displayed Aw values ranging from 0.59 to 0.82 [21]. In the present study, all honey samples showed high water activity (greater than 0.6), which means that stingless bee honey is susceptible to microbial fermentation. Indeed, osmophilic yeasts can grow at Aw values above 0.6 [22].

SBH is more fluid and displays higher moisture content than *A. mellifera* honey [8]. Here, the moisture content of SBH ranged from 19.47% to 36.31% (*w*/*w*). There was a significant difference (*p* < 0.05) in moisture content between the samples from the Atlantic Forest and the Cerrado zones and between the samples from the Caatinga and Cerrado zones. The honey samples MF2 (Cerrado) and MM2 (Atlantic Forest) showed the lowest and highest moisture contents, respectively. The MF2 result may be due to the influence of the climate condition of the Cerrado. During the dry season, relative air humidity can reach low levels (about 9–11%) similar to those of desert regions. Even during the rainy season, high temperatures and a decrease in air relative humidity may eventually occur [23]. SBH produced in different regions of the semi-arid northeastern region of Brazil (Caatinga zone) displayed moisture content values ranging from 23.17% to 28.9% (*w*/*w*) [24,25,26]. Biluca et al., (2016), reported that SBH from the Atlantic Forest zone showed high moisture content (23.1% to 43.5%, *w*/*w*) [27]. The climate of this region is classified as tropical humid [28], which could explain the moisture levels of SBH collected in this biogeographical zone. Similarly, SBH collected in different Ecuadorian regions showed moisture contents ranging from 22% to 30% [29]. The mean annual humidity in Ecuador can range from 65% to 85% [30]. Taken together, these findings suggest that relative air humidity can influence the moisture content of SBH. 

Among the SBH samples investigated in the present study, MM2 and MF2 displayed TSS values of 61.33 and 75.75° Brix, the lowest and the highest values, respectively. In addition, there was a significant difference between samples obtained from the Atlantic Forest and Cerrado zones and those obtained from the Caatinga and Cerrado zones. Previous studies conducted by Brazilian [25,31] and Malaysian [32] research groups demonstrated TSS values similar to those determined here. TSS is closely related to the moisture and sugar content of honey, representing an important indicator of adulteration. In addition, honey produced by stingless bees exhibits lower TSS values than honey produced by *A. mellifera* [33].

Low pH and high acidity values contribute to honey stability and shelf life due to their impact on microbial growth. In addition, these parameters have a strong impact on honey taste diversity. In the present study, all SBH samples showed an acidic character typical of honey, with pH values ranging from 3.06 to 4.78, and free and total acidity values ranging from 17.26 to 130.79 mEq/kg and 71.04 to 299.42 mEq/kg, respectively (Table 2). In addition, we observed that most samples differed significantly (*p* < 0.05) from one another in their free and total acidity content. However, the range of variation was similar to those previously reported for Brazilian SBH [5,19,27]. SBH produced in other countries also has a higher acidic character. Thai SBH exhibited total acidity values ranging from 440 to 592 mEq/kg [8]. Moreover, high free acidity values were reported for SBH collected in the Ecuadorian Amazon (mean value of 318 mEq/kg) [33]. 

Brown pigments and HMF are generated by honey heating or prolonged storage (Maillard reaction) which may lead to important color changes [34]. The present SBH samples showed low brown pigment and HMF content ranging from 0.05 to 0.12 and “not detected” to 12.64 mg.kg^−1^, respectively (Table 2). The low amounts of HMF in SBH are expected and have been attributed to the high moisture and acidity content of this honey in addition to the predominance of fructose. These properties contribute to the inhibition of HMF formation in honey [33]. Color evaluation with the Pfund scale showed that the SBH samples ranged from ≤ 8.00 mm (water white) to 46.72 mm (extra light amber). The predominance of water-like colors in SBH samples collected in distinct biogeographical zones draws attention. Darker honey samples (amber-like colors) were reported by Sant’ana et al. (2020) [31] and de Sousa et al. (2016) [26] for SBH collected in the Brazilian semiarid region. Honey color is influenced by several factors, including climate conditions, botanical origin, harvest time, and degree of honey maturation [32].

### 3.2. Phenolic Content and Antioxidant Capacity of SBH

Table 3 shows that TPC and TFC ranged from 16.3 to 62.33 mg GAE/100 g and 5.39 to 27.22 mg QE/100 g, respectively. The SCA1 and MS2 samples, both collected in the Caatinga zone, had the highest and the lowest TPC and TFC values, respectively. The antioxidant capacity of SBH was evaluated by FRAP and ABTS•+ scavenging assays. As shown in Table 3, SBH displayed FRAP values ranging from 79.53 to 215.07 µmol Fe^+2^/100 g. In the ABTS assay, SBH samples displayed values ranging from 37.84% to 54.95%. Recently, Carina Biluca et al., (2021), reported TPC values of 14 honey samples of six different Brazilian SB ranging from 11.01 to 38.92 mg GAE/100 g [35]. In a previous report, the same research group demonstrated the antioxidant capacity of Brazilian *T. angustula* honey with a FRAP value of 734.5 µmol Fe^+2^/100 g [36]. Few studies have demonstrated the radical scavenging ability of SBH using ABTS•+. Majid et al., (2020), showed that methanol and aqueous extracts of Malaysian SBH displayed ABTS•+ scavenging values ranging from 18.77% to 65.02% and 15.61 to 65.77%, respectively [37]. These values are similar to those obtained in the present study.

### 3.3. Correlation Analysis of Physicochemical Properties and Antioxidant Activity

As expected, strong positive correlations were obtained between some physicochemical parameters such as moisture content and water activity (r = 0.934), and between free and total acidity (r = 0.810) (Table S1). A positive and significant linear correlation was observed between color intensity and TPC (r = 0.654) and between color and TFC (r = 0.866) content of SBH. Moniruzzaman et al., (2013), reported that the color intensity of honey was a consistent parameter that specified the existence of pigments with antioxidant activity, including carotenoids and flavonoids [38]. In the present study, the TPC of Brazilian SBH from different biogeographical zones was positively correlated with TFC (r = 0.705). No significant correlations were found between antioxidant activity and TPC and TFC in SHB samples collected in different biogeographical zones of Brazil (Table S1). These results indicate that the antioxidant activity is not solely dependent on the phenolic and flavonoid content, with other antioxidant compounds present in honey also possibly being involved [32]. The antioxidant capacity of honey could be the result of the combined activity of a narrow range of compounds, including phenolic compounds, peptides, organic acids, and possibly other minor components.

### 3.4. Mineral Profile of SBH

In the present study, we used the TXRF technique to investigate the mineral composition of SBH. The method was validated in terms of TXRF measurements, quantitative analysis, and accuracy (Table S2). TXRF analysis showed a good equivalence of the concentration values of the elements present in the certified sample. The relative errors ranged from 1% to 14%, attesting to the accuracy of the technique.

The results of total concentration of mineral elements in samples of SBH from different biomes are listed in Table 4. Statistically significant differences were found for most minerals investigated. The order of average mineral concentration was K > Ca > Fe > Cu > P > Cl > Mn > Zn > Ni > Cr > Sr in the SBH samples from the Atlantic Forest; K > P > Ca > Cl > Cu > Fe > Mn > Sr > Zn > Cr > Ni in the Caatinga samples, and K > Ca > P > Cu > Zn > Fe > Mn > Sr > Ni > Cu in the Cerrado samples. The mineral profile analysis of honey is important because of the presence of metallic species which can have a nutritional or toxic effect depending on the metal present and/or the amount ingested [39]. The difference in the concentrations of metals in honey can also be attributed to biological, chemical, and physical factors and the existence of industrial contamination, thus providing important information about the region where the honey is produced [40].

Quantitatively, K (67.35–679.50 µg/g) > Ca (21.47–165 µg/g) > P (2.59–100.19 µg/g) were the main minerals found in honey from stingless bees in the biomes studied. This is consistent with the findings reported by Biluca et al., (2016), which demonstrated that K (27.3 to 448 mg/100 g) and Ca (1.12 to 35.2 mg/100 g) were the most abundant minerals in samples of SBH [27]. These results also corroborate those described by Pucholobek et al., (2022), who also observed that Ca (4.3 to 363.8 µg/g) is one of the most abundant minerals in the honey of stingless bees from Brazil [39]. These same authors observed a strong correlation between the concentration of minerals in honey and the type of soil in the study region. Plants accumulate metallic elements and this accumulation is influenced by the mineral character of the soil. In the present study, the Atlantic Forest honey had a higher content of Ca and Fe than the honey from the Cerrado and Caatinga, especially considering Fe. On the other hand, the Cerrado and Caatinga honey had the highest P content. The biogeographical regions where the samples were collected have soil types with compositional characteristics that might influence the amounts of these metals in honey. Indeed, these characteristics may explain the differences between regions for the elements P, K, Ca, Fe, Zn, Rb, and Se. It is noteworthy that the Fe content detected was about eight times higher in honey samples from the Atlantic Forest region than in samples from the other regions. This result was not observed for any of the other minerals, which showed differences of much smaller orders of magnitude. The Atlantic Forest has a variety of soil types, but with a predominance of soil rich in iron oxide (Latosol/Nitosol), which may influence the amounts of this metal in honey [39].

### 3.5. Discriminant Analysis of SBH

The fingerprinting of SBH is shown as Supplementary Materials (Figures S1–S3). Ion masses within the *m/z* range of 50–1000, physicochemical features, and mineral content were analyzed statistically to discriminate honey samples using the producing stingless bee species or the biogeographical origin. Minor ions responsible for group separation are shown in Figures S1–S3.

**Figure 1:**
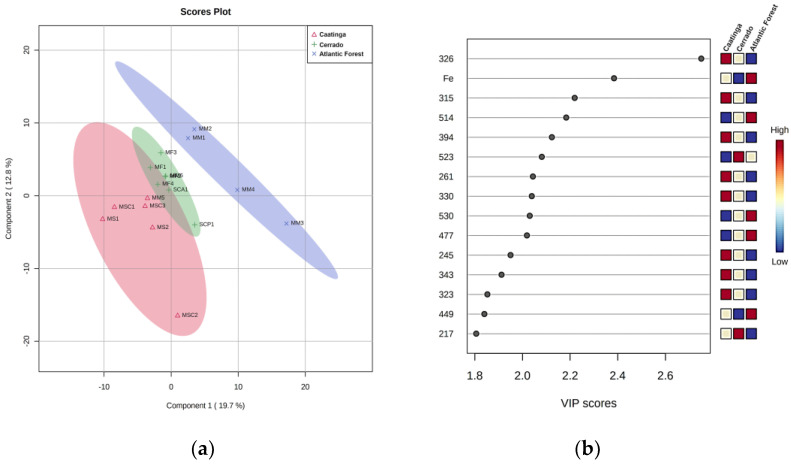
Discriminant analysis of SBH based on Brazilian biogeographical zones. (**a**) PLS-DA score plots; and (**b**) VIP score of physicochemical parameters, mineral profile, antioxidant capacity, and mass fingerprint analysis of SBH.

Different species of stingless bees may have specific preferences for botanical individuals when exploiting food sources [41]. Thus, we initially evaluated the influence of stingless bee species on the chemical and physicochemical features of honey. Figure S4 shows that bee species has little impact on sample discrimination. Nonetheless, when samples were categorized by their biogeographical origin, better separation of the groups was achieved. PLS-DA analysis (Figure 1a) shows little overlap between the Cerrado and Caatinga ellipsoids, while the Atlantic Forest group did not overlap with any other group. These results demonstrate that the chemical and physicochemical features of SBH are mainly influenced by their respective biogeographical origin.

The main separation factors between groups were the *m/z* 326, 315, 514, 394, 523, 261, 330, 530, 477 ions, and Fe content. Each of these factors showed VIP scores > 2, which confirmed their weight for group separation (Figure 1b). The *m/z* 326, 315, 394, 291, and 330 ions had a high weight in the Caatinga group and a low weight in the Atlantic Forest group. On the other hand, iron content had a high weight in the Atlantic Forest group and a low weight in the Cerrado group, while the *m/z* 514, 530, and 477 ions had a high weight in the Atlantic Forest group and a low weight in the Caatinga group. Univariate statistical analysis of iron content confirmed its influence on group separation, with a significant difference (*p* < 0.05) between samples from the Cerrado and Atlantic Forest, and between samples from the Caatinga and Atlantic Forest. These results suggest that iron content could be used to indicate the biogeographical origin of the sample. Interestingly, the iron content of 88 SBH was positively correlated with the soil types of areas where the honey samples were collected. High amounts of iron were detected in samples collected from areas of typical Atlantic Forest flora, where iron-rich soil is predominant [39]. Here, we did not observe a significant difference in iron content between samples from the Cerrado and Caatinga. The Cerrado soil is acidic, poor in nutrients, and with a low capacity to retain water [42], characteristics that are very close to those of crystalline and sedimentary caatingas (two subgroups of the Caatinga zone) [43].

The other variables identified as VIP in multivariate analysis did not show significant differences in univariate analyses. This can be explained by the difference between univariate and multivariate methods. A variable-by-variable (univariate) analysis, such as ANOVA with post hoc tests, uses few criteria to determine significant differences between samples, requiring these criteria to be extremely strong to determine separations into groups. An analysis model that uses variable information and correlations with other variables (multivariate), such as the PLS-DA, allows the identification of many more criteria to determine significant differences between samples and determine separations into groups.

## 4. Conclusions

This study demonstrated that Brazilian SBH has diverse physicochemical properties, antioxidant capacity, and chemical and mineral profiles. Despite the phenolic and flavonoid contents, no correlation was observed between them and the antioxidant capacity, suggesting that other compounds contribute to the antioxidant properties of SBH. The mineral profile displayed high amounts of K, Ca, and P in all samples. PLS-DA analysis revealed that the composition of SBH is influenced by the biogeographical zone of collection. In addition, Fe content was identified as the main factor responsible for SBH separation into the biogeographical zone groups. Taken together, the present results highlight the mineral content, especially the amounts of Fe, as an important factor for SBH discrimination.

## Figures and Tables

**Table 1:** Details of stingless bee species providing samples.

Biogeographical Zones	Bee Species	Popular Name	Year (Season)	Sample Identifier
Atlantic Forest	*Melipona mondury*	Uruçu Amarela	2017 (autumn)	MM1
	*Melipona mondury*	Uruçu Amarela	2017 (autumn)	MM2
	*Melipona mondury*	Uruçu Amarela	2017 (autumn)	MM3
	*Melipona mondury*	Uruçu Amarela	2018 (summer)	MM4
	*Melipona quadrifasciata*	Mandaçaia	2018 (spring)	MQ1
Caatinga	*Melipona subnitida*	Jandaíra	2018 (autumn)	MS1
	*Melipona subnitida*	Jandaira	2018 (winter)	MS2
	*Melipona scutellaris*	Uruçu Nordestina	2018 (winter)	MSC1
	*Melipona scutellaris*	Uruçu Nordestina	2018 (summer)	MSC2
	*Melipona scutellaris*	Uruçu Nordestina	2018 (summer)	MSC3
	*Melipona mondury*	Uruçu Amarela	2018 (summer)	MM5 (6)
	*Scaptotrigona aff. postica*	Tubi	2018 (winter)	SCA1
Cerrado	*Melipona fasciculata*	Tiúba	2018 (winter)	MF1
	*Melipona fasciculata*	Tiúba	2018 (winter)	MF2
	*Melipona fasciculata*	Tiúba	2018 (winter)	MF3
	*Melipona fasciculata*	Tiúba	2018 (spring)	MF4
	*Scaptotrigona polysticta*	Benjoi	2018 (winter)	SCP1
	*Melipona mondury*	Uruçu Amarela	2018 (summer)	MM6 (5)

**Table 2:** Physicochemical parameters of honey produced by stingless bees from different Brazilian biogeographical zones.

Biogeogr. Zone	Samples	Aw	Moisture (%)	TSS (°Brix)	pH	Total Acidity (meq/Kg)	Free Acidity (meq/Kg)	Brown Pigment	HMF (mg/Kg)	Pfund (mm)	Color Name
Atlantic Forest	MM1	0.75 ± 0.00 ^c,d^	30.43 ± 0.37 ^c,d^	67.65 ± 0.35 ^g^	3.24 ± 0.10 ^f^	71.04 ± 0.55 ^k^	59.74 ± 0.99 ^f^	0.05 ± 0.00 ^d^	0.38 ± 0.08 ^d^	23.82 ± 0.00 ^d^	White
	MM2	0.75 ± 0.00 ^c^	36.31 ± 0.09 ^a^	61.33 ± 0.13 ^i^	3.57 ± 0.02 ^b^	81.79 ± 1.65 ^j^	67.11 ± 1.87 ^e^	0.12 ± 0.00 ^a^	5.16 ± 1.17 ^b^	46.72 ± 0.00 ^a^	Extra light amber
	MM3	0.73 ± 0.00 ^f,g^	28.55 ± 0.70 ^e,f^	69.33 ± 0.45 ^e,f^	3.26 ± 0.03 ^e,f^	143.86 ± 2.77 ^i^	83.80 ± 1.86 ^c^	0.12 ± 0.00 ^a^	nd	28.77 ± 0.00 ^c^	White
	MM4	0.74 ± 0.01 ^d,e^	30.79 ± 0.65 ^c^	69.05 ± 1.13 ^e,f,g^	3.40 ± 0.00 ^c,d^	240.67 ± 0.00 ^c^	75.26 ± 0.00 ^d^	0.07 ± 0.00 ^b,c,d^	nd	≤8.00	Water white
	MQ1	0.81 ± 0.00 ^a^	35.79 ± 0.10 ^a^	61.92 ± 0.13 ^i^	-	-	-	0.07 ± 0.01 ^b,c,d^	nd	0.00	Water white
Caatinga	MS1	0.72 ± 0.00 ^g,h^	27.96 ± 0.10 ^e,f,g^	70.08 ± 0.13 ^e^	3.37 ± 0.00 ^c,d,e^	204.91 ± 0.00 ^g^	50.80 ± 0.00 ^h^	0.07 ± 0.02 ^b,c,d^	nd	≤8.00	Water white
	MS2	0.73 ± 0.00 ^e,f^	28.83 ± 0.00 ^d,e,f^	69.25 ± 0.00 ^e,f^	3.26 ± 0.05 ^e,f^	211.09 ± 0.00 ^f^	42.50 ± 0.00 ^i^	0.06 ± 0.02 ^c,d^	1.05 ± 0.00 ^d^	≤8.00	Water white
	MSC1	0.77 ± 0.00 ^b^	32.90 ± 0.20 ^b^	65.00 ± 0.23	3.26 ± 0.11 ^e,f^	299.42 ± 0.00 ^a^	130.79 ± 0.00 ^a^	0.12 ± 0.00 ^a^	0.15 ± 0.00 ^d^	15.39 ± 0.00 ^e^	Extra white
	MSC2	0.74 ± 0.00 ^e,f^	28.13 ± 0.18 ^e,f,g^	70.08 ± 0.13 ^e^	3.47 ± 0.00 ^b,c^	201.97 ± 0.00 ^g^	33.35 ± 0.00 ^j^	0.09 ± 0.01 ^a,b,c,d^	nd	15.27 ± 0.00 ^f^	Extra white
	MSC3	0.73 ± 0.00 ^e,f^	29.16 ± 1.36 ^c,d,e,f^	67.67 ± 1.32 ^g^	4.78 ± 0.00 ^a^	179.11 ± 0.00 ^h^	17.26 ± 0.00 ^k^	0.09 ± 0.01 ^a,b,c,d^	1.03 ± 0.00 ^d^	≤8.00	Water white
	MM5 (6)	0.75 ± 0.00 ^c^	29.52 ± 0.95 ^c,d,e^	67.92 ± 0.13 ^f,g^	3.54 ± 0.00 ^b^	220.17 ± 0.00 ^e^	58.13 ± 0.00 ^f^	0.10 ± 0.03 ^a,b,c^	3.56 ± 0.00 ^c^	≤8.00 ^h^	Water white
	SCA1	0.71 ± 0.00 ^i^	25.92 ± 0.00 ^h^	72.25 ± 0.00 ^c^	3.06 ± 0.00 ^g^	288.71 ± 0.00 ^b^	120.52 ± 0.00 ^b^	0.12 ± 0.02 ^a^	3.43 ± 0.00 ^c^	40.78 ± 0.00 ^b^	Extra light amber
Cerrado	MF1	0.68 ± 0.00 ^j^	23.87 ± 0.10 ^i^	74.17 ± 0.13 ^b^	3.46 ± 0.00 ^b,c^	227.45 ± 0.00 ^d^	53.51 ± 0.00 ^g^	0.07 ± 0.01 ^b,c,d^	nd	≤8.00	Water white
	MF2	0.65 ± 0.00 ^k^	19.47 ± 0.63 ^j^	75.75 ± 0.22 ^a^	3.30 ± 0.00 ^d,e,f^	218.32 ± 0.00 ^e^	51.69 ± 0.00 ^g,h^	0.07 ± 0.00 ^b,c,d^	nd	≤8.00	Water white
	MF3	0.67 ± 0.00 ^j^	23.13 ± 0.21 ^i^	75.08 ± 0.13 ^a,b^	-	-	-	0.07 ± 0.01 ^b,c,d^	nd	≤8.00	Water white
	MF4	0.67 ± 0.00 ^j^	23.40 ± 0.00 ^i^	74.92 ± 0.13 ^a,b^	-	-	-	0.07 ± 0.01 ^b,c,d^	nd	≤8.00	Water white
	SCP1	0.71 ± 0.00 ^i^	26.61 ± 0.10 ^g,h^	71.75 ± 0.23 ^c,d^	-	-	-	0.11 ± 0.01 ^a,b^	12.64 ± 0.00 ^a^	13.11 ± 0.00 ^g^	Extra White
	MM6 (5)	0.72 ± 0.00 ^h^	27.72 ± 0.00 ^f,g^	70.50 ± 0.00 ^d,e^	-	-	-	0.08 ± 0.03 ^a,b,c,d^	0.45 ± 0.00 ^d^	≤8.00	Water white

- = not analyzed; nd = not detected. Values are expressed as mean ± standard deviation. ^a–k^ = different superscript letters in the same column denote significant differences (ANOVA and Tukey, *p* < 0.05).

**Table 3:** Contents of phenolic compounds, total flavonoids, and antioxidant activity of stingless bee honey of different species from different biogeographical zones of Brazil. TPC and TFC results are expressed as mean concentration (mg/100 g FW) ± standard deviation. The FRAP result is expressed as the mean value of µmol Fe^2+^ ± standard deviation of fresh honey samples. The ABTS result is expressed as the mean percentage of free radical scavenging power ± standard deviation of fresh honey samples.

Biogeographical Zone	Samples	TPC (mg GAE/100 g FW)	TFC (mg QE/100 g FW)	Antioxidant Activity	
				FRAP (µmol Fe^2+^/100 g FW)	ABTS (%)
Atlantic Forest	MM1	47.11 ± 1.39 ^a,b,c^	16.70 ± 0.32 ^b^	110.85 ± 4,42 ^b,c^	38.22 ± 1.47 ^b,c^
	MM2	44.46 ± 1.16 ^a,b,c,d^	15.02 ± 0.18 ^b,c^	114.49 ± 9.38 ^b,c^	40.08 ± 2.07 ^b,c^
	MM3	35.69 ± 2.45 ^b,c,d,e^	13.53 ± 0.04 ^c,d^	84.87 ± 8.53 ^c^	51.61 ± 8.24 ^a,b^
	MM4	46.74 ± 5.75 ^a,b,c^	9.93 ± 0.38 ^f,g^	212.16 ± 8.84 ^a^	39.82 ± 1.70 ^b,c^
	MQ1	29.80 ± 4.50 ^c,d,e,f^	9.04 ± 0.38 ^f,g,h,i^	-	-
	MS1	32.99 ± 9.44 ^b,c,d,e,f^	6.99 ± 0.63 ^j,k^	108.95 ± 1.18 ^b,c^	39.82 ± 2.20 ^b,c^
	MS2	16.30 ± 4.26 ^f^	5.39 ± 1.07 ^k^	-	-
	MSC1	25.26 ± 1.66 ^d,e,f^	8.89 ± 1.22 ^f,g,h,i^	-	39.20 ± 3.42 ^b,c^
	MSC2	22.24 ± 18.18 ^e,f^	10.55 ± 0.21 ^e,f^	118.71 ± 23.28 ^b,c^	48.74 ± 6.30 ^a,b,c^
	MSC3	39.29 ± 7.23 ^b,c,d,e^	8.33 ± 0.21 ^g,h,i,j^	215.07 ± 7.27 ^a^	54.95 ± 11.88 ^a^
	MM5(6)	36.03 ± 6.17 ^b,c,d,e^	7.91 ± 0.04 ^h,i,j^	143.96 ± 15.80 ^b^	43.71 ± 4.79 ^a,b,c^
	SCA1	62.33 ± 4.33 ^a^	27.22 ± 3.04 ^a^	-	-
Cerrado	MF1	39.86 ± 6.88 ^b,c,d,e^	9.26 ± 0.07 ^f,g,h^	88.70 ± 0.19 ^c^	44.20 ± 5.49 ^a,b,c^
	MF2	37.98 ± 6.00 ^b,c,d,e^	7.66 ± 0.10 ^h,i,j^	84.53 ± 2.55 ^c^	37.84 ± 3.24 ^c^
	MF3	50.95 ± 4.59 ^a,b^	7.39 ± 0.07 ^i,j^	79.53 ± 2.55 ^c^	40.08 ± 3.21 ^b,c^
	MF4	26.68 ± 2.19 ^d,e,f^	6.67 ± 0.60 ^j,k^	88.56 ± 5.91 ^c^	38.22 ± 1.30 ^b,c^
	SCP1	34.30 ± 11.23 ^b,c,d,e,f^	12.13 ± 0.07 ^d,e^	140.03 ± 3.34 ^b^	45.09 ± 6.68 ^a,b,c^
	MM6 (5)	36.41 ± 9.89 ^b,c,d,e^	7.91 ± 0.18 ^h,i,j^	-	-

- = not analyzed. Values are expressed as mean ± standard deviation. ^A–k^ = different superscript letters in the same column denote significant differences (ANOVA and Tukey test, *p* < 0.05). TPC = total phenolic content; TFC = total flavonoid content.

**Table 4:** Mineral profile of SBH from different Brazilian biogeographical zones. The results are expressed as mean concentration (µg/g FW) ± standard deviation. Minimum and maximum concentrations are shown in parenthesis.

Mineral	Atlantic Forest	*Caatinga*	*Cerrado*
P	3.54 ± 0.56	49.20 ± 29.81	20.19 ± 14.36
	(3.01–4.06) ^a^	(4.60–100.19) ^b^	(2.59–40.75) ^b^
Cl	1.71 ± 1.69	4.20 ±1.46	nd
	(0.69–0.16) ^a^	(2.54–6.41) ^a^	
K	411.58 ± 107.74	234.22 ± 109.23	127.03 ± 44.57
	(249.01–679.50) ^a^	(79.73–419.23) ^a^	(67.35–177.99) ^b^
Ca	91.65 ± 48.98	39.18 ± 15.72	31.84 ± 6.20
	(44.28–165.00) ^a^	(21–47–57.28) ^b^	(24.45–39.75) ^b^
Cr	0.27 ± 0.22	0.26 ± 0.13	0.19 ± 0.01
	(0.04–0.55) ^a^	(0.15–0.48) ^a^	(0.18–0.19) ^a^
Mn	1.24 ± 0.91	0.90 ± 0.48	0.56 ± 0.16
	(0.47–2.66) ^a^	(0.37–1.75) ^a^	(0.36–0.77) ^a^
Fe	8.71 ± 4.41	1.32 ± 0.47	0.84 ± 0.10
	(1.68–12.92) ^a^	(0.96–2.32) ^b^	(0.73–0.97) ^c^
Ni	0.61 ± 0.45	0.22 ± 0.08	0.21 ± 0.01
	(0.01–0.99) ^a^	(0.13–0.30) ^a^	(0.20–0.21) ^a^
Cu	3.76 ± 3.80	3.86 ± 1.73	2.31 ± 0.67
	(0.34–9.82) ^a^	(1.70–6.72) ^a^	(1.66–3.33) ^a^
Zn	0.87 ± 0.38	0.36 ± 0.22	0.88 ± 0.29
	(0.44–1.36) ^a^	(0.10–0.73) ^b^	(0.49–1.23) ^a^
Rb	1.22 ± 0.41	0.54 ± 0.36	0.52 ± 0.01
	(0.85–1.59) ^a^	(0.20–1.08) ^a,b^	(0.51–0.52) ^b^
Sr	0.10 ± 0.02	0.38 ± 0.26	0.26 ± 0.08
	(0.08–0.11) ^a^	(0.15–0.81) ^b^	(0.19–0.33) ^b^

Nd = not detected. Different superscript letters on the same line indicate significant differences (ANOVA and Tukey test, *p* < 0.05).

## Data Availability

The data that support the findings of this study are available from the corresponding author upon reasonable request.

## Supplementary Materials

The following supporting information can be downloaded at: https://www.mdpi.com/article/10.3390/foods12010180/s1, Table S1: Correlation matrix between physicochemical and antioxidant activity of samples of stingless bee honey from different geographical regions of Brazil; Table S2: Results of the standard reference material 1577b; Figure S1: Mass spectra of stingless bee honey collected in the Atlantic Forest biogeographical zone; Figure S2: Mass spectra of stingless bee honey collected in the Caatinga biogeographical zone; Figure S3: Mass spectra of stingless bee honey collected in the Cerrado biogeographical zone; Figure S4: Discriminant analysis of SBH based on bee species.
