# Supplementary material for: Discriminant Analysis of Brazilian Stingless Bee Honey Reveals an Iron-Based Biogeographical Origin
Source: Foods. 2023 Jan 1;12(1):180. doi: 10.3390/foods12010180 (PMC9818887; doi:10.3390/foods12010180)
Supplement: Supplementary file 1 [file foods-12-00180-s001.zip › foods-2065618-supplementary.pdf]

foods

Supplementary

Discriminant analysis of Brazilian stingless bee honey reveals an iron-based biogeographical origin

Flavia C. Lavinas[1], Brendo A. Gomes[2], Marcos V. T. Silva[1], Renata M. Nunes[1], Suzana G. Leitão[3], Mirian R. L. Moura[3], Rosineide C. Simas[4,5], Carla S. Carneiro[3,*] and Igor A. Rodrigues[3,*]

[1] Programa de Pós-graduação em Ciências Farmacêuticas, Faculdade de Farmácia, Universidade Federal do Rio de Janeiro, Rio de Janeiro, 21941-902, Brazil

[2] Programa de Pós-graduação em Biotecnologia Vegetal, Universidade Federal do Rio de Janeiro, Rio de Janeiro, 21941-902, Brazil

[3] Laboratorio de Cromatografia e Espectrometria de Massas (LaCEM), Universidade Federal de Goiás, Goiânia, 74.690-900, Brazil

[4] Escola Presbiteriana Mackenzie, Escola de Engenharia, Instituto Mackenzie, Higienópolis, São Paulo, 01302-907, Brazil;

[5] Departamento de Produtos Naturais e Alimentos, Faculdade de Farmácia, Universidade Federal do Rio de Janeiro, Rio de Janeiro, 21941-902, Brazil

* Correspondence: CSC, carlacarneiro@pharma.ufrj.br; IAR, igor@pharma.ufrj.br

Table S1. Correlation matrix between physicochemical and antioxidant activity of samples of stingless bee honey from different geographical regions of Brazil.

	Aw	TSS	Mois	BP	pH	FA	TA	HMF	Color	TPC	TFC	FRAP	ABTS
**Aw**	**1**												
**TSS**	**-0.864**	**1**											
**Mois**	**0.903**	**-0.963**	**1**										
**BP**	0.291	**-0.361**	**0.330**	**1**									
**pH**	0.115	-0.220	0.134	0,003	**1**								
**FA**	0.209	-0.153	0.205	**0.534**	**-0.709**	**1**							
**TA**	-0.177	**0.407**	**-0.344**	**0.538**	-0.429	**0.810**	**1**						
**HMF**	-0.344	-0.163	0.129	0.417	0.485	-0.311	-0.174	**1**					
**Color**	-0.511	-0.145	0.184	**0.514**	-0.208	0.159	-0.295	0.417	**1**				
**TPC**	-0.202	0.099	-0.061	0.132	-0.032	0.303	-0.083	0.385	**0.637**	**1**			
**TFC**	0.005	-0.031	0.078	**0.401**	-0.305	**0.532**	-0.076	0.209	**0.866**	**0.705**	**1**		
**FRAP**	**0.422**	-0.312	0.359	-0.114	**0.636**	-0.289	0.227	-0.300	-0.323	0.194	-0.226	**1**	
**ABTS**	0.194	-0.072	0.064	0.336	**0.559**	-0.342	0.009	-0.264	-0.002	-0.126	-0.072	0.299	**1**

The bold numbers indicate a statistically significant correlation ($p < 0.05$).

foods

Table S2. Results of the standard reference material 1577b.

Elements	Calculated Concentration (μg/g)*	Certified Value (μg/g)	Relative error (%)
P	1010.2 ± 36.9	1100	8
S	874.2 ± 28.2	785	11
Cl	304.6 ± 15.8	278	9
Ca	103.8 ± 11.1	116	10
Mn	11.1 ± 2.4	10.5	5
Fe	209.8 ± 4.3	184	14
Cu	139.6 ± 3.3	160	13
Zn	115.0 ± 3.0	127	9
Rb	13.9 ± 2.3	13.7	1

*Mean ± standard deviation (n=13).

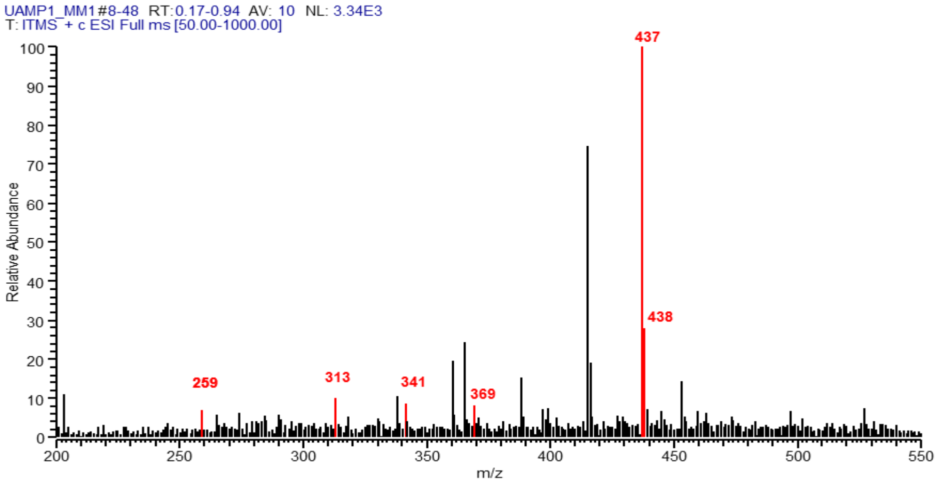

(a)

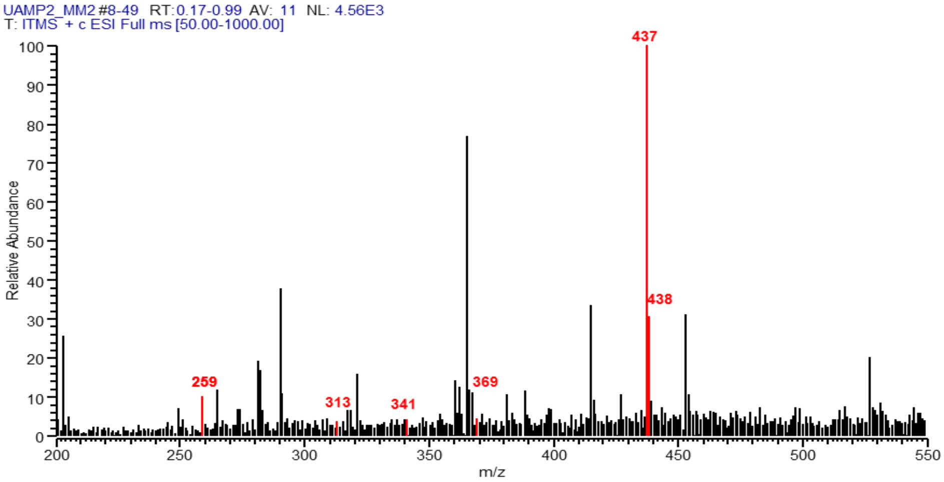

(b)

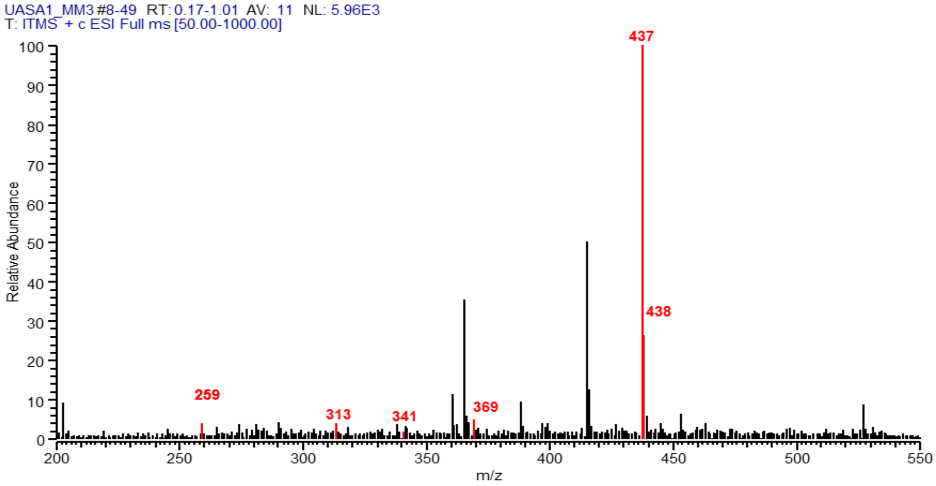

(c)

Figure S1. Mass spectra of stingless bee honey collected in the Atlantic Forest biogeographical zone. (a) MM1; (b) MM2; (c) MM3. Ion masses responsible for samples' separation into groups (VIP score > 2) are highlighted in red.

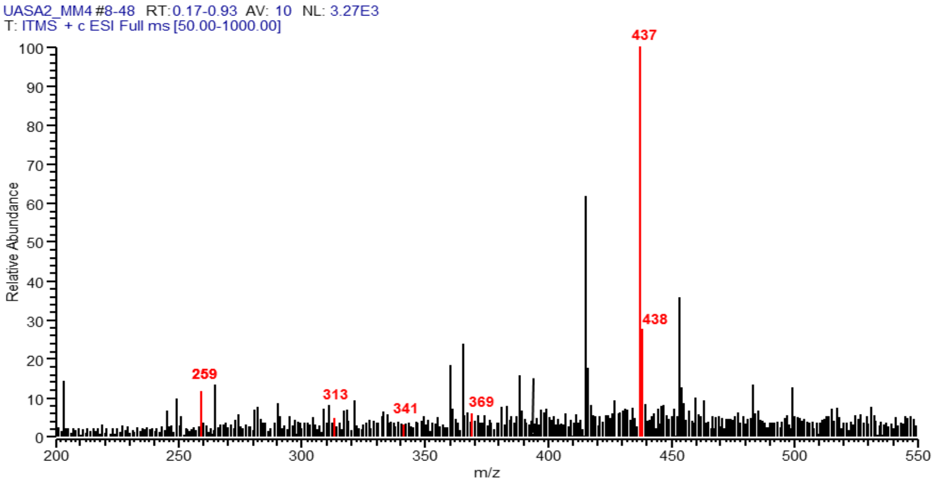

(d)

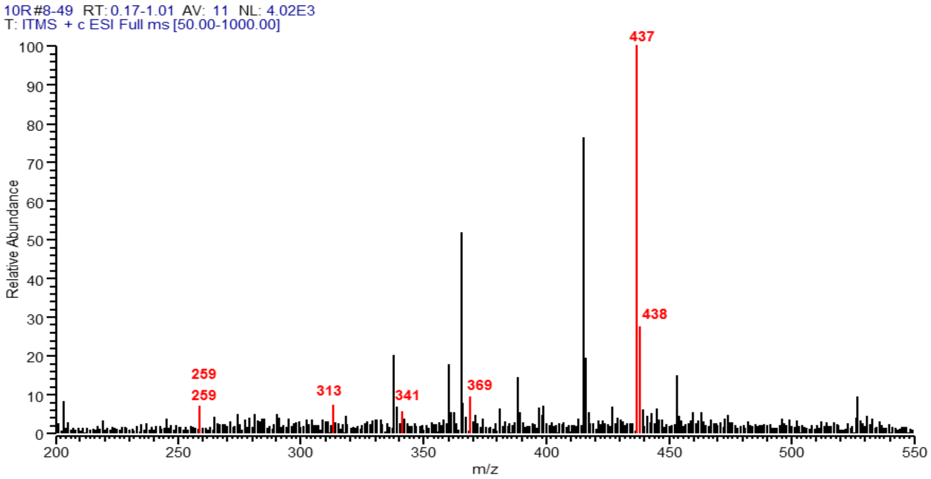

(e)

Continuation of Figure S1. Mass spectra of stingless bee honey collected in the Atlantic Forest biogeographical zone. (d) MM4; (e) MQ1. Ion masses responsible for samples' separation into groups (VIP score > 2) are highlighted in red.

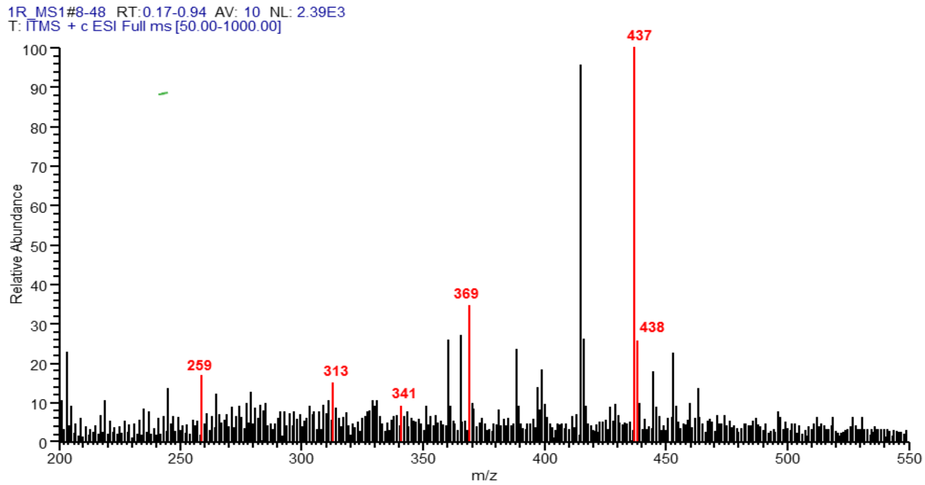

(a)

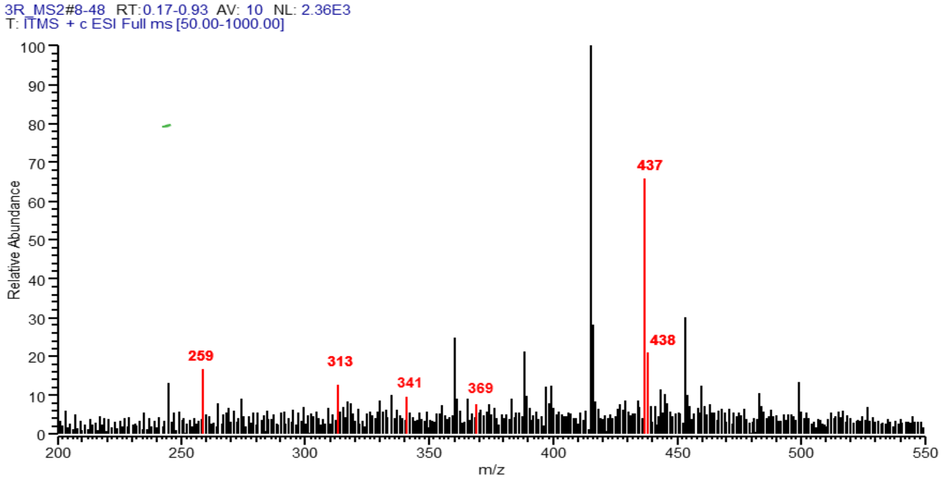

(b)

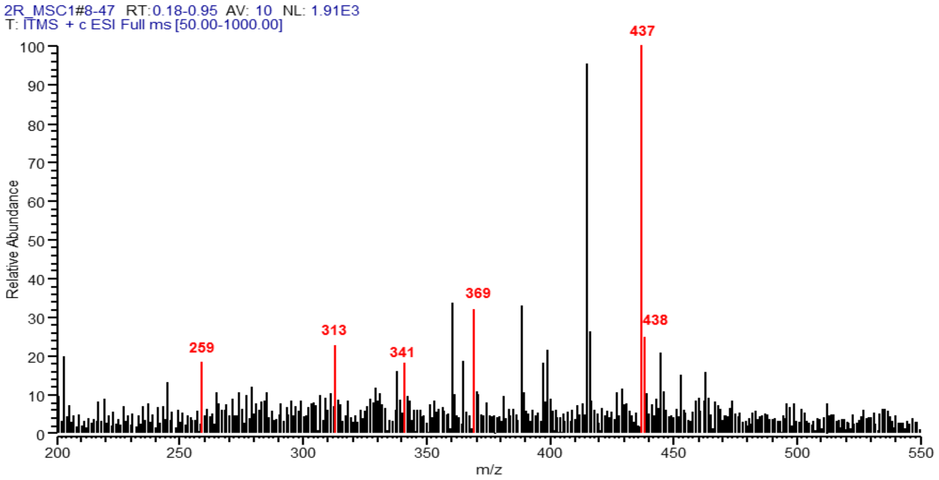

(c)

Figure S2. Mass spectra of stingless bee honey collected in the *Caatinga* biogeographical zone. (a) MS1; (b) MS2; (c) MSC1. Ion masses responsible for samples' separation into groups (VIP score > 2) are highlighted in red.

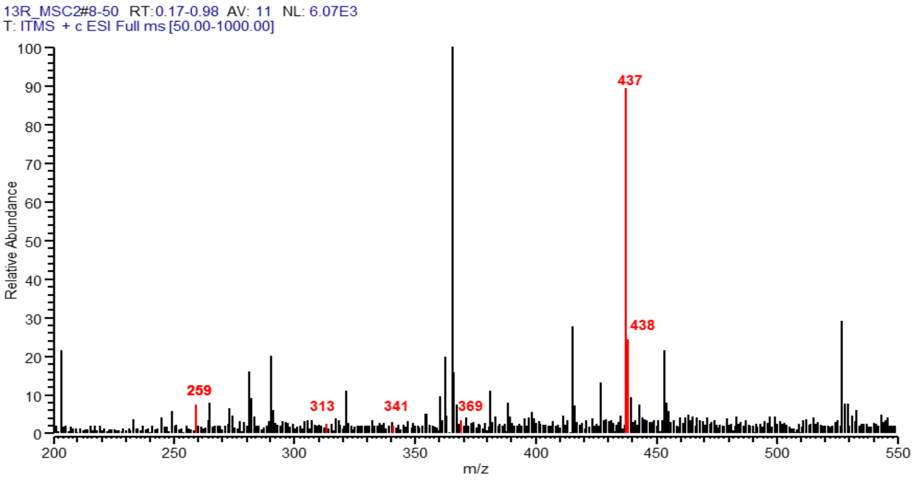

(d)

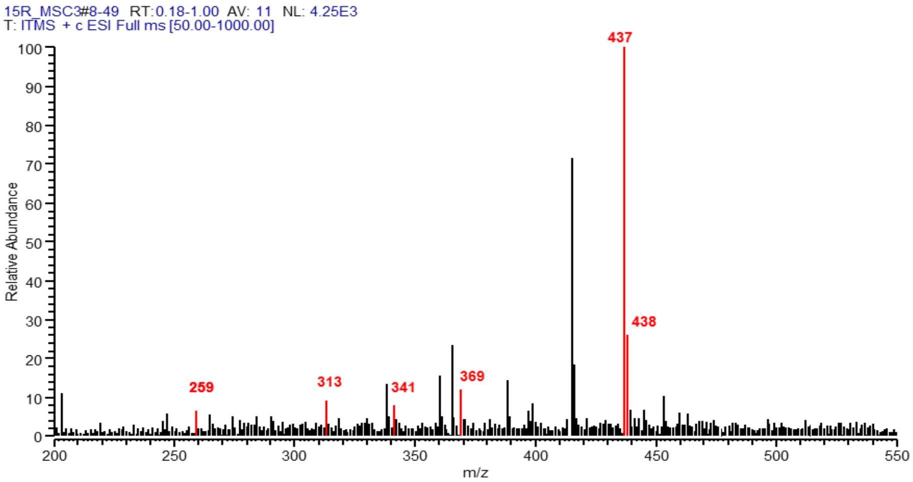

(e)

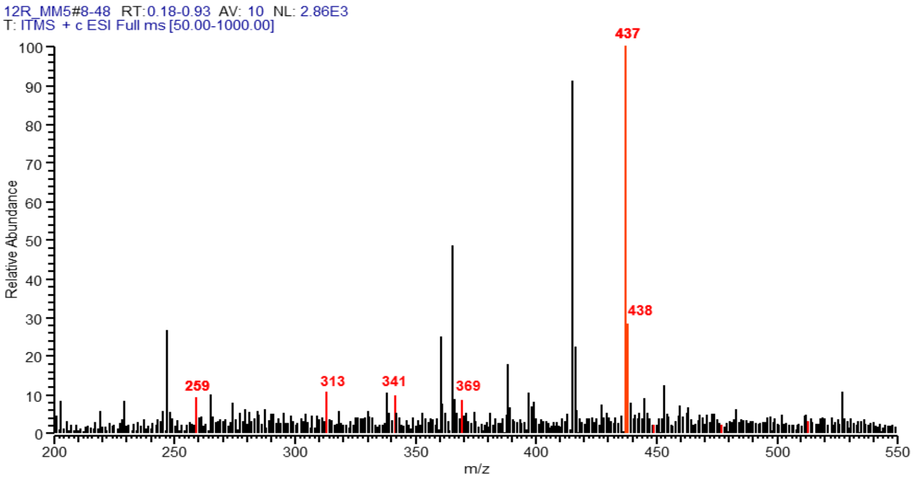

(f)

Continuation of Figure S2. Mass spectra of stingless bee honey collected in the Caatinga biogeographical zone. (d) MSC2; (e) MSC3; (f) MM5. Ion masses responsible for samples' separation into groups (VIP score > 2) are highlighted in red.

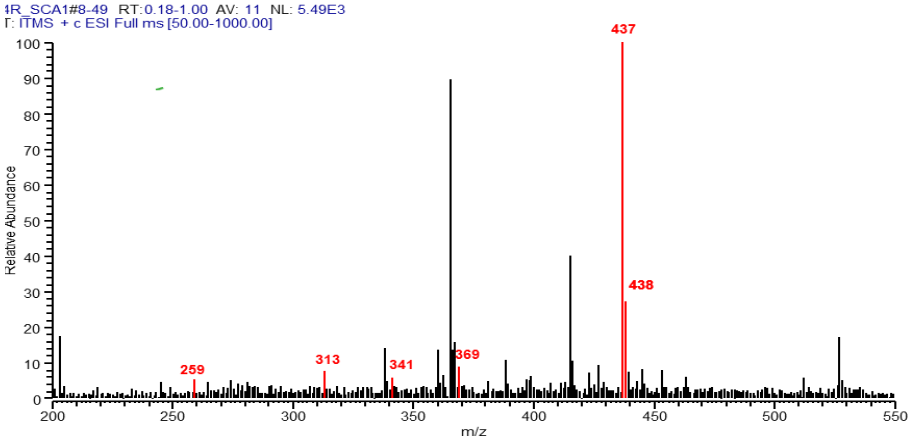

(g)

Continuation of Figure S2. Mass spectra of stingless bee honey collected in the *Caatinga* biogeographical zone. (g) SCA1. Ion masses responsible for samples' separation into groups (VIP score > 2) are highlighted in red

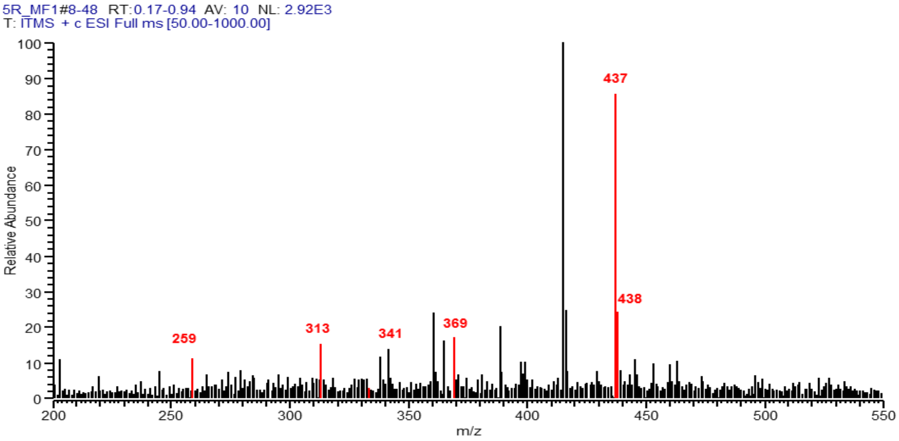

(a)

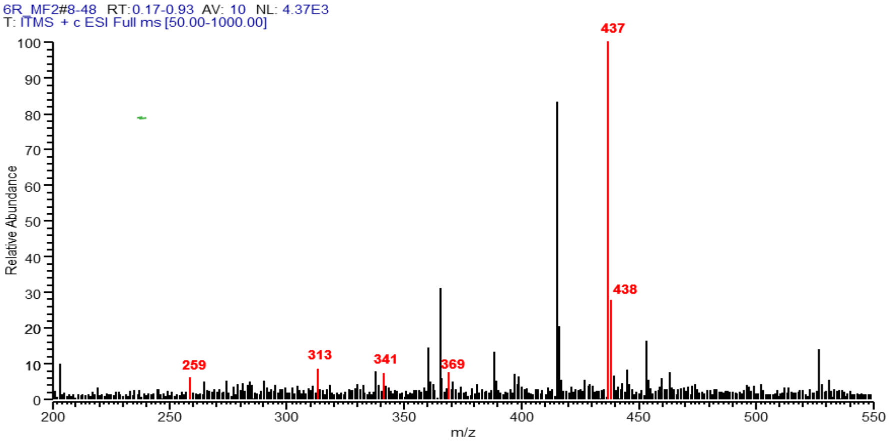

(b)

Figure S3. Mass spectra of stingless bee honey collected in the *Cerrado* biogeographical zone. (a) MF1; (b) MF2. Ion masses responsible for samples' separation into groups (VIP score > 2) are highlighted in red.

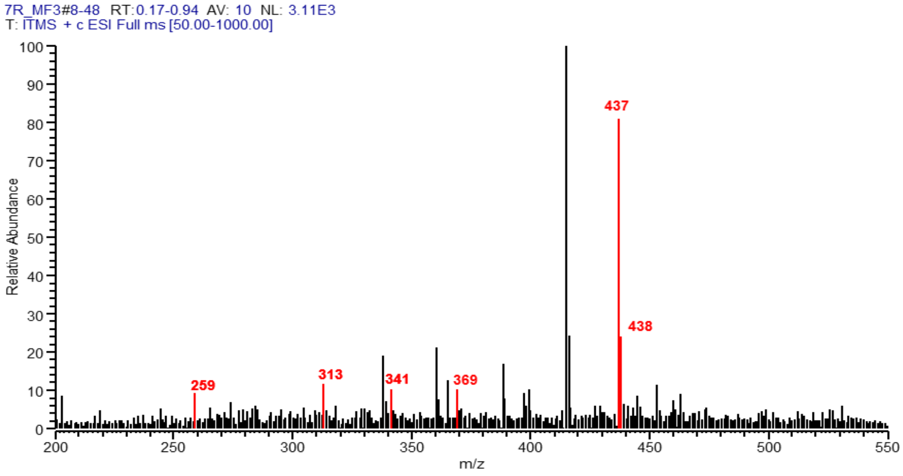

(c)

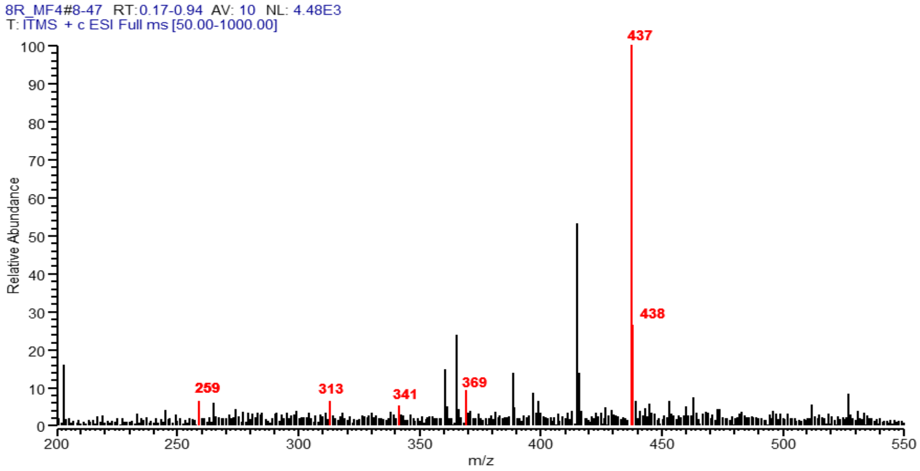

(d)

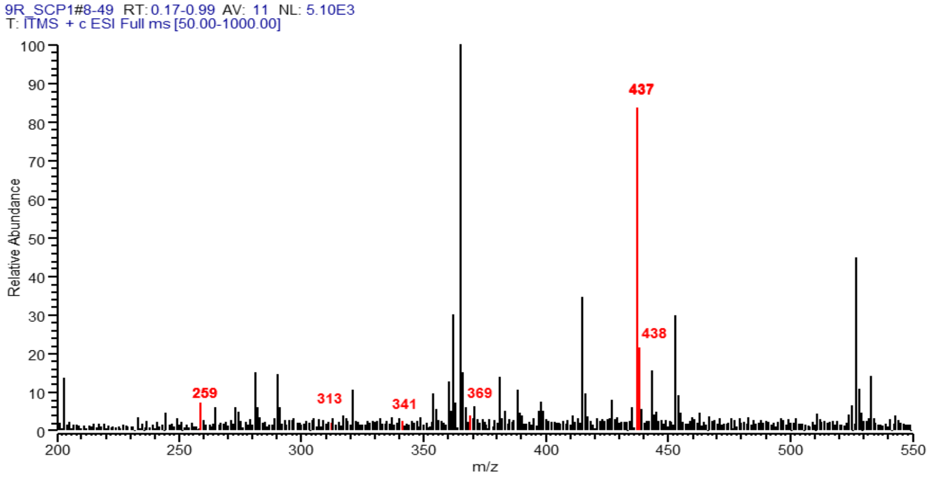

(e)

Continuation of Figure S3. Mass spectra of stingless bee honey collected in the *Caatinga* biogeographical zone. (c) MF3; (d) MF4; (e) SCP1. Ion masses responsible for samples' separation into groups (VIP score > 2) are highlighted in red.

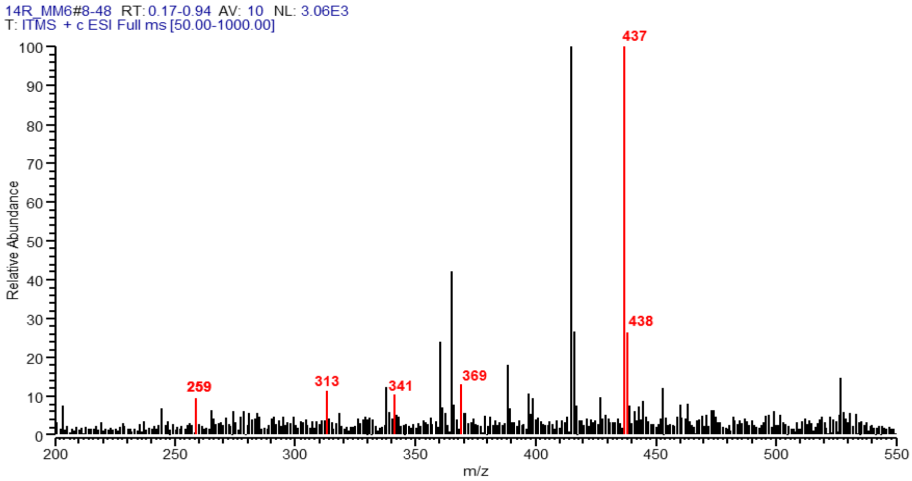

(f)

Continuation of Figure S3. Mass spectra of stingless bee honey collected in the *Caatinga* biogeographical zone. (f) MM6. Ion masses responsible for samples' separation into groups (VIP score > 2) are highlighted in red.

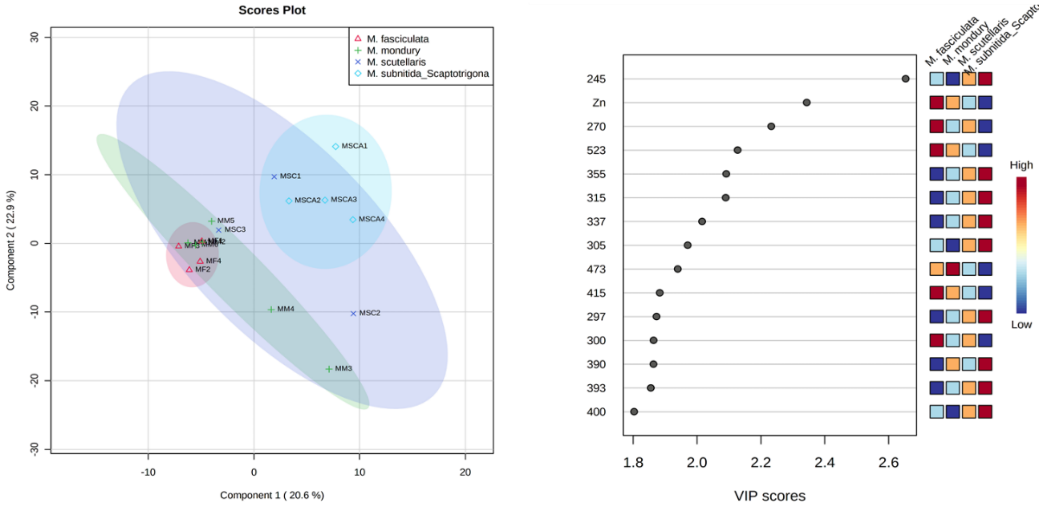

(a) (b)

Figure S4. Discriminant analysis of SBH based on bee species. PLS-DA score plots (a) and VIP score (b) of physicochemical parameters, mineral profile, antioxidant capacity, and mass fingerprint analysis of stingless bee honey.
